# Comparison of Outcomes between Endoscopic and Transcleral Cyclophotocoagulation

**DOI:** 10.3390/vision1040024

**Published:** 2017-11-06

**Authors:** Robert Beardsley, Simon K. Law, Joseph Caprioli, Anne L. Coleman, Kouros Nouri-Mahdavi, Jean-Pierre Hubschman, Steven D. Schwartz, JoAnn A. Giaconi

**Affiliations:** 1 Stein Eye Institute, Los Angeles, CA 90095, USA; 2 Greater Los Angeles Veterans Affairs, Los Angeles, CA 90073, USA

**Keywords:** cyclophotocoagulation, endoscopic cyclophotocoagulation, transcleral cyclophotocoagulation, cycloablation, cyclodestruction

## Abstract

*Importance*: Traditionally cyclophotocoagulation has been reserved as a treatment of last resort for eyes with advanced stage glaucoma, but increasingly it is offered to eyes with less severe disease. Endoscopic approaches in particular are utilized in increasing numbers of patients despite only a small number of publications on its results. *Objective*: The purpose of this study was to compare the efficacy and safety of endoscopic and transcleral cyclophotocoagulation (ECP and TCP) procedures in eyes with refractory glaucomas. *Design, Setting, and Participants*: A chart review was performed on consecutive patients who underwent ECP and TCP at a tertiary ophthalmology care center between January 2000 and December 2010. Cases with fewer than 3 months of follow-up or that had concurrent pressure reducing procedures were excluded. The main outcome measures examined were intraocular pressure (IOP), number of glaucoma medications, best corrected visual acuity (BCVA), additional glaucoma procedure required, and complications. *Main Outcomes and Measures*: Forty-two eyes (42 patients) that underwent ECP and forty-four eyes (44 patients) that underwent TCP were identified. The TCP group had a statistically higher mean age (71.2 ± 16.7 vs. 58.1 ± 22.9 years, respectively), larger proportion of neovascular glaucoma (40.9% vs. 16.7%), worse initial BCVA (logMAR 2.86 vs. 1.81), and higher preoperative IOP (45.3 vs. 26.6 mmHg) than the ECP group. At 12 months follow-up, the mean IOP difference between groups was not statistically significant, although the change in IOP from baseline to 12 months was greater for the TCP group (*p* = 0.006). The rates of progression to no light perception (NLP) and phthisis bulbi were significantly higher amongst TCP eyes than ECP eyes (27.2% vs. 4.8%, *p* = 0.017, and 20.5% vs. 0%, *p* = 0.003, respectively). Of these eyes that progressed, a majority had neovascular glaucoma (NVG). Corneal decompensation was the most frequent complication following ECP (11.9%). *Conclusions and Relevance*: In patients with preoperative BCVA of 20/400 or better, overall complication rates (cystoid macular edema, exudative retinal detachment, inflammation, cornea decompensation) were higher after ECP than with TCP. In refractory glaucomas in a real world setting (not a trial), TCP was more frequently used in ischemic eyes. TCP was associated with a higher rate of progression to phthisis bulbi and loss of light perception than ECP. However, ECP was associated with a clinically significant rate of corneal decompensation. These outcomes likely were related to the severity of underlying ocular diseases found in these eyes.

## 1. Introduction

Laser cyclophotocoagulation is a surgical procedure that treats the ciliary processes and thereby reduces aqueous humor production in order to decrease intraocular pressure (IOP). Using a diode laser, ablation of the ciliary processes can be carried out either as endoscopic cyclophotocoagulation (ECP) or as transcleral cyclophotocoagulation (TCP). Advantages of cyclophotocoagulation lie in refractory glaucomas where outflow procedures and medications have been utilized unsuccessfully, and in the case of TCP, in patients who cannot go to the operating room. 

Laser cyclophotocoagulation in the form of TCP has generally been reserved for eyes with limited visual potential due to significant rates of vision loss and phthisis bulbi reported in the literature [1,2]. However, there are also studies of TCP in eyes with good visual acuity that have not shown these concerning complication rates [3,4,5,6,7]. For eyes with good vision, it has been suggested that ECP may be advantageous over TCP because ECP provides a more targeted destruction of the ciliary epithelium, sparing the ciliary process blood flow [8]. The lower reported incidence of phthisis and hypotony following ECP may be attributed to the lack of choroidal blood flow disruption. These factors may make ECP preferable for eyes with neovascular glaucoma (NVG), a disease in which there is a higher rate of hypotony and phthisis bulbi following TCP as compared to treatment of other glaucoma types [9]. However, direct comparison of ECP and TCP is limited in the literature because of differences in patient populations, glaucoma subtype, and disease severity of patients undergoing ECP and TCP. 

In this study, we present a large consecutive case series of all ECP and TCP procedures performed at a single institution for a variety of glaucomas. The purpose of this study is to compare the outcomes and complications associated with each procedure. Additionally, we evaluate the two procedures in eyes with better initial visual acuity (20/400 or better) and in eyes with NVG. 

## 2. Materials and Methods 

Medical records of all patients who underwent ECP or TCP between January 2000 and December 2010 at the Jules Stein Eye Institute were retrospectively reviewed. University of California, Los Angeles Institutional Review Board approval was obtained for this study. This research was adherent to the tenets of the Declaration of Helsinki. Patients were excluded if postoperative follow-up was shorter than 3 months or if a concurrent pressure reducing surgery was performed. One eye per patient was enrolled. If both eyes of a patient had the same type of cyclophotocoagulation procedure (ECP or TCP) and qualified, the first eye that received the cycloablation procedure was enrolled. 

ECP was performed with the EndoOptiks E2 system 810 nm diode laser (Endo Optiks, Inc., Little Silver, NJ, USA). ECP treatment was delivered by inserting the treatment probe through a temporal clear corneal paracentesis or through a pars plana sclerotomy. TCP was performed with the IRIS Oculight 810 nm with G-Probe (Iridex, Inc., Mountain View, CA, USA). 

Preoperative data collected included demographic characteristics, systemic diagnoses and medications, ocular diagnoses, intraocular pressure (IOP), glaucoma medications, best-corrected visual acuity (BCVA), and previous ocular surgeries. Intraoperative data collected included total intraoperative energy applied in Joules for TCP, extent of treatment in degrees for ECP, and intraoperative complications. TCP energy (in Joules) delivered was calculated by multiplying the number of laser burns by the duration (seconds) and power (Watts). ECP was performed at 25 mJ on a continuous mode setting; only circumferential extent of treatment was reported in operative reports. Postoperatively, BCVA, IOP, and number of glaucoma medications were collected at 1 month (±2 weeks), 3 months (±4 weeks), 6 months (±2 months), 12 months (±2 months), and each year (±2 months) thereafter. Additionally, post-operative complications, and repeat surgical and cyclophotocoagulative interventions were recorded throughout the entire follow-up period. 

Early complications were defined as those that occurred within one month following surgery, while late complications were defined as those that developed after 1 month. Hypotony was defined as an IOP at or below 5 mmHg without phthisis, and phthisis was defined as intraocular disorganization regardless of IOP level. Corneal decompensation was defined as persistent stromal edema greater than 3 months duration in a previously clear cornea. Uveitis was defined as persistent anterior chamber reaction greater than 1 month in duration. 

BCVA was modeled as the negative logarithm of the reciprocal of the minimum angle of resolution (logMAR) for the purposes of statistical analysis. LogMAR values of 2.3, 2.9, 3.2, and 3.5 were assigned to counting fingers, hand motion, light perception, and no light perception visions, respectively, according to the grading scheme of the World Glaucoma Association [10].

Outcome measurements included intraocular pressure (IOP), number of glaucoma medications, best corrected visual acuity (BCVA), additional glaucoma procedure required, and complications. The Wilcoxon signed rank test was used to compare outcomes within groups. The Wilcoxon rank sum test, Fisher exact test, and Student *t*-test were used to compare differences in outcomes between study groups. Statistical analysis was performed with SAS software version 9.1 (SAS, Inc., Cary, NC,USA). *p*-values ≤ 0.05 were considered statistically significant. Mean values were presented with their standard deviations (±S.D.).

## 3. Results

A total of 42 eyes of 42 patients treated by ECP and 44 eyes of 44 patients treated by TCP met inclusion criteria. Mean follow-up times between the two groups were similar (19.4±17.3 months after ECP and 17.8 ± 18.7 months after TCP). Patients who underwent TCP had different preoperative characteristics than those who underwent ECP. (Table 1) Patients who underwent TCP were older (mean age: 71.2 ± 16.7 vs. 58.1 ± 22.9 years, *p* = 0.003, respectively) and had higher preoperative IOP (45.3 vs. 26.6 mmHg, *p* = 0.001). In addition, the TCP group had a greater proportion of neovascular glaucoma (40.9% vs. 16.7%, *p* = 0.05), but a smaller proportion of patients that had prior known glaucoma surgeries (20.5% vs. 92.9%, *p* = 0.0001).

Preoperative visual acuity in the TCP group was worse than in the ECP group, and the mean vision in both groups was poor (logMAR 2.86 vs. 1.81, respectively, *p* = 0.0001, which is worse than 20/800 for both groups). No light perception (NLP) vision prior to cyclophotocoagulation was more prevalent in TCP than in ECP patients (50.0% vs. 2.4%, *p* = 0.0001). Conversely, the ECP group had a higher proportion of patients with BCVA of 20/400 or better than the TCP group (42.9% vs. 15.9%). 

On average, 257.8 ± 70.9° of the pars plicata was treated by ECP. ECP was performed via a clear-cornea approach in 38 eyes and via the pars plana in four eyes. In the TCP group, the mean treatment time was 1.7 s, the power was 1984 mW, and 21.3 applications were given on average. Treatment was over 360° in all but two cases and the three and nine o’clock positions where posterior ciliary arteries emerge forward were avoided.

### 3.1. Intraocular Pressure Control

Intraocular pressure and need for glaucoma medications were significantly reduced in both the ECP and TCP groups. (Table 2) At the 12 month follow-up, mean IOP was reduced from 26.6 ± 10.9 mmHg at baseline to 19.0 ± 9.4 mmHg in the ECP group (*p* = 0.013) and from 45.3 ± 15.4 at baseline to 22.2 ± 12.7 mmHg in the TCP group (*p* < 0.0001). The final mean IOP at 12 months of follow-up was similar between the groups (19.0 [ECP] and 22.2 mmHg [TCP]) despite a large difference in the baseline IOPs (difference between groups for change in IOP from baseline to 12 months, *p* = 0.006). The mean number of glaucoma medications required was reduced from 3.1 to 2.3 in the ECP group (*p* < 0.001) and from 3.0 to 2.0 in the TCP group (*p* < 0.001). 

Additional glaucoma procedures were performed for inadequate IOP control in 16/42 ECP eyes (38.1%) and in 16/44 TCP eyes (36.4%). In the ECP group, repeat cyclophotocoagulation was performed in 6/42 eyes (14.2%) (two had repeat ECP and four eyes received subsequent TCP). A glaucoma drainage device was placed in 9/42 (21.4%) of the ECP eyes. Of the ECP cohort that required additional glaucoma surgery, the initial extent of treatment was 276.9 ± 56.2° (slightly greater than the average extent of treatment for the entire group). In the TCP group, seven eyes received a glaucoma drainage device, one eye underwent trabeculectomy, one had ECP, and seven had repeat TCP. Those TCP eyes that required further treatment had slightly lower initial treatment energy of 62.2 ± 30.9 Joules compared to the group average. 

### 3.2. Visual Acuity

The differences between preoperative and 12-month follow-up average BCVA was not significant in the ECP group (p = 0.57) but mildly significant in the TCP group (*p* = 0.03) (Table 2). Of the 63 eyes between both groups with preoperative sight, six eyes progressed to NLP vision. Progression to NLP was more common in the TCP group, 6/21 eyes (28.5%), than in the ECP group, 2/41 (4.9%) (*p* = 0.017) (Table 3) The six eyes that went NLP after TCP had baseline visions of HM (n = 1) to LP (n = 5) and progressed to NLP vision between postoperative month 1 and year 4. All eyes had NVG: four were secondary to central retinal vein occlusion, one from diabetic retinopathy, and one due to intraocular tumor. The two ECP eyes that progressed to NLP had starting visions of 20/200 and CF. The eye starting with 20/200 vision carried a diagnosis of Stickler’s syndrome and suffered an intraoperative choroidal hemorrhage during pars plana ECP and progressed to NLP within 1 month of surgery. The eye with CF vision at baseline had a diagnosis of NVG due to central retinal artery occlusion and maintained CF vision through the 6 month follow-up but was NLP at the subsequent 12 month time point, presumably due to progressive ischemia from the artery occlusion. 

### 3.3. Complications

Intraocular complications were reported rarely. One eye, as noted above, suffered an acute choroidal hemorrhage during ECP via a pars plana approach. Early and late postoperative complications are summarized in Table 3. The rate of early postoperative complications between ECP and TCP were similar. Retinal detachment developed in one eye of each group; an exudative retinal detachment was seen after ECP and a rhegmatogenous retinal detachment developed in one TCP eye. Late complications were encountered more commonly in the TCP group. Phthisis bulbi developed in nine TCP eyes (20.5%) compared to zero ECP eyes (*p* = 0.003). However, corneal decompensation in previously clear corneas was more frequent in ECP. It was seen in five ECP eyes (11.4%) and in one TCP eye (2.5%). Sympathetic ophthalmia was not encountered. 

### 3.4. Subgroup Analysis: Neovascular Glaucoma

There were 25 eyes that had NVG; seven eyes underwent ECP and 18 eyes underwent TCP. Initial BCVA was generally poor in both ECP and TCP groups (logMAR 2.99, 3.25, respectively, *p* = 0.13, [2.9 = HM]). BCVA at 12 months follow-up worsened in both groups, *p* = 0.57 for the change in vision for the ECP group and *p* = 0.03 for the TCP group. Vision was NLP prior to cyclophotocoagulation procedure in one eye that underwent ECP and in 10 eyes that underwent TCP. Loss of preoperative vision to NLP occurred in one of six eyes (16.7%) in the ECP group and in four of eight eyes (50%) in the TCP group (*p* = 0.30).

Mean preoperative IOP of the NVG eyes was greater in the TCP group than in the ECP group (47.8 mmHg, 32.1 mmHg, respectively, *p* = 0.02). IOP at 12 months follow-up was lower in the TCP than in the ECP group (14.6 mmHg, 29.3 mmHg, respectively, *p* = 0.05) (Table 2).

Hypotony at final follow-up was seen in six eyes (33.3%) with NVG in the TCP group versus none in the ECP group. Progression to phthisis occurred in 7/18 eyes (37.8%) treated with TCP but in none in the ECP group (*p* = 0.13). There were no other significant differences in either early or late complications between the two groups (Table 3).

### 3.5. Subgroup Analysis: Patients with BCVA 20/400 or Better

There were 25 eyes with BCVA of 20/400 or better. ECP was performed on 18 and TCP on seven of these better sighted eyes. There were no significant differences between the two groups with regards to preoperative and postoperative BCVA or change of BCVA after cyclophotocoagulation procedure at 12 months follow-up. A loss of two or more lines of vision occurred in 7/18 (38.8%) eyes in the ECP group (with one eye losing vision to NLP) and in one out of seven eyes (14.3%) in the TCP group, however a comparison failed to reach statistical significance. The eye that lost all vision in the ECP group was the previously mentioned Stickler syndrome eye with intraoperative choroidal hemorrhage. The reduction of IOP failed to reach significance in either group (ECP *p* = 0.54 and TCP *p* = 0.25) likely due to small numbers (Table 2). There was a significantly higher rate of early and late postoperative complications seen in the ECP group, such as cystoid macular edema, exudative retinal detachment, choroidal hemorrhage, inflammation, and corneal decompensation (Table 3). However, none of these reached statistical significance due to the small sample size of the TCP group. 

## 4. Discussion

In this retrospective cases series of ECP and TCP, it is evident that both procedures may achieve significant pressure reduction. Patients who underwent TCP were older with worse preoperative vision and higher IOP, and more eyes had NVG than in the ECP cohort. The different preoperative characteristics of patients who underwent ECP and TCP reflect the traditional practice of using TCP on eyes with refractory glaucomas with little visual potential. This treatment bias stems from an association with vision loss in a substantial number of eyes treated by cyclophotocoagulation of the ciliary processes [1,2,11]. In the subgroup analysis of eyes with NVG, TCP was observed to have a high rate of postoperative complications, including phthisis bulbi and loss of light perception. In the small number of eyes with BCVA of 20/400 of better, ECP was observed to have a high rate of corneal decompensation and overall postoperative complications. The number of eyes and difference in loss to follow-up in the subgroup analyses limit the ability to draw strong conclusions comparing the two procedures in these subgroups. 

The effectiveness of IOP reduction with TCP and ECP in our series is consistent with the literature. An IOP reduction of ≥30% from baseline has been reported in 12.3–66% of TCP procedures [11]. In our series, 63.6% of TCP cases and 57.1% of ECP cases showed at least a 30% reduction in IOP. Also, in our series a final IOP of 5-21 mmHg was seen in 34.1% of TCP and 71.4% of ECP procedures, whereas in the literature at 1 year this final range of IOP was seen in 54.0–92.7% of TCP and 17–94% ECP cases, according to one review [11]. Reasons for such varying rates of success amongst studies include different patient populations and different treatment protocols. The amount of energy delivered has been correlated to treatment success in some studies [12,13,14], although not in all [4,15,16]. Our total average energy administered of 71.8 J fell within the range of previous TCP reports from 46.6 to 155.2 J [11]. Other factors found to influence treatment results include preoperative IOP level [4], age [17,18,19], glaucoma type [17,18,19], history of previous surgery [18,19,20], and pigmentation [13,21,22].

Complications in our groups of TCP and ECP are similar to those seen in the literature, except for a greater incidence of corneal decompensation than previously reported for ECP. The literature reports a low incidence of serious complications following ECP, although it has been noted that ECP complications are higher in eyes with NVG and refractory glaucomas [23,24,25,26,27]. In our ECP group, we encountered fibrinoid reactions, retinal detachment, choroidal hemorrhage, vitritis, cystoid macular edema, corneal decompensation, and progression to NLP vision. Corneal decompensation requiring further treatment (penetrating keratoplasty or keratoprosthesis) was the most frequent adverse event in the ECP group, occurring in five of 42 eyes (11.9%); one case developed decompensation acutely and four progressed to decompensation after 1 month. Francis et al. similarly reported a 12% rate of corneal or corneal graft edema in 25 eyes following ECP [24]. Phthisis bulbi was exclusively seen in eyes that underwent TCP in our study, although it has been reported in the literature following ECP [25,26]. Loss of vision was experienced by eyes in both treatment groups, but was more frequent in the TCP group. Progression to NLP vision was significantly greater in the TCP group, which overall contained eyes with more advanced disease, and five of the six eyes that ended up NLP were LP prior to treatment. Of the two ECP eyes that progressed to NLP, ECP may or may not have contributed to the ultimate causes of NLP, which were a choroidal hemorrhage in an eye with Stickler’s syndrome and ischemia in an eye with NVG secondary to central retinal artery occlusion. 

A series by Ramli et al. demonstrated that eyes with NVG are at much higher risk for hypotony after TCP than eyes with other forms of glaucoma [9]. We evaluated our subset of eyes with NVG and found that 11.1% of cases with NVG progressed to hypotony after TCP. A total of 38.8% (7/18) became phthisical. Our average energy level, 75.4 J (median 69 J, and range 5–185 J) was similar to the 83.3 ± 31.7 J used in Ramli’s study. Of the sighted eyes undergoing TCP, half progressed to NLP. Interestingly, of the seven cases of NVG treated by ECP none progressed to phthisis or hypotony, but one did progress to NLP vision. The follow up in the NVG ECP group was 9.2 months shorter than in the NVG TCP group, and longer follow-up might have allowed for the development of phthisis or hypotony. Animal studies have demonstrated less disorganization of the ciliary architecture and vasculature following ECP, as well as partial reperfusion of the processes within 1 month of ECP that was not seen after TCP [8,28], The less destructive nature of ECP may be of benefit in NVG eyes that have a compromised vasculature. However, there may not be a totally safe procedure for vasculopathic eyes. Even after tube shunts there is a high rate of progression to NLP in NVG eyes of 24% to 31%, and a tube implantation does not affect the eye’s vasculature [29,30]. We observed that ECP was better tolerated in our small group of NVG eyes , however, it is possible that eyes with less severe ischemia were selected for ECP treatment in our cohort. A larger sample is required to confirm this observation. 

Additionally, given reports using ECP and TCP as a primary treatment of glaucoma and in eyes with good visual potential [3,4,5,6,31], we examined eyes with 20/400 or better vision to see if either treatment had a detrimental effect on vision, IOP, and complications. In a retrospective review by Rotchford et al. of ECP in eyes with an initial median BCVA of 20/30, a loss of two lines of BCVA was seen in 30% of eyes. The authors could not attribute the loss of vision to the procedure rather than to the underlying glaucoma given the mean follow up of 5 years. Similar rates of vision loss over 5 years have been seen secondary to glaucoma progression in studies utilizing other types of glaucoma surgery [32,33]. In our subgroup of eyes with 20/400 or better vision, 38.8% of ECP eyes and 14.3% of TCP eyes lost >2 lines of vision. Progression to NLP occurred in one ECP eye but no TCP eyes. In refractory glaucomas undergoing ECP, reported complication rates are higher than rates seen in milder glaucomas treated by ECP: vision loss of at least two lines has been reported as high as 6% [34], hypotony in 8% [27], and phthisis in up to 3% of cases [25]. Although the number of eyes in this study’s subgroup is very small, these rates of vision loss at 1 year may call for caution in treating eyes with refractory glaucomas and better vision. 

This study is limited by its retrospective nature. There is an acknowledged bias in treatment recommendations, as the more severely diseased eyes tended to undergo TCP rather than ECP. However, the entire group of eyes falls into the severe stage of glaucoma or even end stage glaucoma. A prospective study can address these deficiencies, and be appropriately powered to detect a difference in IOP outcomes between the two procedures. Additionally, the rate of complications is likely underestimated in this study, both due to the retrospective design and the clarity of media of some of the included eyes, which may have limited complete clinical evaluation of the posterior segment. Complications like cystoid macular edema (quoted to occur in up to 4.3%) and fibrin exudates (7.3%) are difficult to detect in eyes with cloudy media [25]. However, rates of vision loss would still be accurately reported even with the retrospective design. Lastly, the subset analyses of NVG and eyes with better vision were small limiting generalizability. Strengths include a relatively large sample size and inclusion of all types of refractory glaucomas, which allows for greater generalizability to what may happen in clinical practice. 

## 5. Conclusions 

In conclusion, each method of cyclophotocoagulation demonstrates an ability to significantly reduce IOP in a variety of advanced glaucomas. The ECP group had a higher corneal decompensation rate but less progression to phthisis and NLP than the TCP group, which conversely had a higher rate of final NLP vision and phthisis. Sympathetic ophthalmia was seen in neither group. This data, although limited due to its retrospective collection bias, may suggest that further investigation of the rate of phthisis bulbi between ECP and TCP is warranted in eyes with neovascular glaucoma. Lastly, these data may suggest caution using ECP for treatment of refractory glaucomas in eyes with usable vision and low corneal endothelial cell counts, as the rate of corneal decompensation here was clinically significant.

## Figures and Tables

**Table 1:** Baseline Characteristics of Subjects.

Subject Characteristics	ECP	TCP	*p* Value *
Total subjects	42	44	
Age in years (Mean ± StdDev)	58.1 ± 22.9	71.2 ± 16.7	0.003 **
Male, No.(%)	28 (66.7)	24 (54.5)	0.28
Right Eye, No.(%)	25 (59.5)	18 (40.9)	0.13
Initial BCVA (logMar)	1.81	2.86	0.0001
Initial NLP eyes, No.(%)	1 (2.4)	22 (50.0)	<0.0001
Blood thinner use (%)	20.5	0.31	
Systemic comorbidity, No.(%)	27 (64.3)	32 (72.7)	0.49
Type of glaucoma, No. (%)			0.0496
Congenital	8 (19.0)	2 (4.5)	
Primary Open Angle	9 (21.4)	6 (13.6)	
Secondary Open Angle	5 (11.9)	2 (4.5)	
Chronic Angle Closure	3 (7.1)	4 (9.1)	
Secondary Angle Closure (traumatic)	2 (4.8)	2 (4.5)	
Secondary Angle Closure (nontraumatic)	6 (14.3)	6 (13.6)	
Secondary Angle Closure (neovascular)	7 (16.7)	18 (40.9)	
Aphakic	1 (2.4)%	3 (6.8)	
Other/Nonspecified	1 (2.4)	1 (2.3)	
Prior glaucoma surgery, No. (%)	29 (92.9)	9 (20.5)	<0.0001
Any prior eye surgery, No.(%)	42 (100)	30 (68.2)	<0.0001
Lens status			0.15
Pseudophakic, No.(%)	21 (50.0)	18 (40.9)	
Aphakic, No.(%)	11 (26.2)	7 (15.9)	
Phakic, No.(%)	10 (23.8)	19 (43.2)	

* All *p*-values were from Fisher exact test unless noted otherwise; ** *t* test; ECP: endoscopic cyclophotocoagulation, TCP: transcleral cyclophotocoagulation, BCVA: best corrected visual acuity, POAG: primary open angle glaucoma, NLP: no light perception, Systemic comorbidities include hypertension, diabetes mellitus, cerebrovascular accident, cancer.

**Table 2:** Intraocular pressure, vision, and medications before and after ECP and TCP as seen in all eyes, neovascular eyes, and eyes with BCVA 20/400 or better.

			Total Cohort					Neovascular Glaucoma Cohort						BCVA ≥ 20/400 Cohort								
		ECP			TCP			ECP			TCP			ECP					TCP			
	n	Mean	*p*-Value	n	Mean	*p*-Value	n	Mean	*p*-Value	n	Mean	*p*-Value	n	Mean		*p*-Value		n		Mean		*p*-Value
**Follow up (mo)**		19.5			17.8			13.6			22.8			18.6						17.4		
**Initial BCVA**	42	1.81		44	2.86	0.0001 *	7	2.99		18	3.25	0.13 *	18	0.62				7		0.51		*p* = 0.39 *
**12 mo. BCVA**	24	1.62		23	2.99		3	3.1		11	3.39		11	0.68				3		0.42		
**Change in BCVA**	24	−0.03	0.57	23	0.09	0.03	3	0.4	1	11	0.19	0.06	11	0.09		0.98		3		−0.1		0.5
**Preoperative IOP (mmHg)**	42	26.6		44	45.3		7	32.1		18	47.8		18	21.8				7		33.4		
**12 month IOP (mmHg)**	23	18.9		23	22.2		3	29.3		11	14.6		10	20.1				3		27.0		
**Change in IOP (mmHg)**	23	−7.7	0.01	23	−22.6	<0.0001	3	−3.0	1	11	−31.8	0.002	10	−1.8		0.54		3		−9.7		0.25
**Initial Medications**		3.1			3			3.1			2.8			3.5						3.1		
**Final Medications**		2.3			2			2.1			1.3			2.3						3.6		
**Repeat Procedure or Surgery, No. (%)**	42	16 (38.1)		44	16 (36.4)		7	1 (14.3)		18	5 (27.8)		18	7 (38.9)				7		4 (57.1)		

IOP: intra-ocular pressure, ECP: endoscopic cyclophotocoagulation, TCP: transcleral cyclophotocoagulation, BCVA: best corrected visual acuity. All *p*-values were from Wilcoxn signed rank test unless otherwise noted in order to compare pre-operative and 12-month outcomes within the ECP or TCP cohorts. * Wilcoxon rank sum test was used to initial BCVA between ECP and TCP in all groups.

**Table 3:** Complications of ECP and TCP as seen in all eyes, neovascular eyes, and eyes with BCVA 20/400 or better.

	Total Cohort			NVG Cohort			BCVA >20/400 Cohort		
	ECP	TCP	*p*-Value *	ECP	TCP	*p*-Value *	ECP	TCP	*p*-Value *
**Sample Size**	42	44		7	18		18	7	
**Acute Adverse Event (<1month), No.**									
Fibrinoid reaction	1	0	0.49	0	0	n/a	1	0	1.0
Corneal decompensation	1	0	0.5	0	0	n/a	0	0	n/a
Post operative cylinder	1	0	0.49	0	0	n/a	1	0	1.0
Persistent AC reaction	2	0	0.24	0	0	n/a	0	0	n/a
Epitheliopathy	1	0	0.49	1	0	1.0	0	0	n/a
Choroidal hemorrhage	1	1	1.0	0	0	n/a	1	0	1.0
Vitritis	1	0	0.49	0	0	n/a	0	0	n/a
Macular Edema	2	0	0.24	0	0	n/a	2	1	1.0
Retinal Detachment	1	1	1.0	0	0	n/a	0	0	n/a
Hyphema	0	1	1.0	0	1	1.0	0	0	n/a
Persistent Lid Edema	0	1	1.0	0	1	1.0	0	0	n/a
Malignant glaucoma	0	1	1.0	0	1	1.0	0	0	n/a
**Late Adverse Event (>1 month), No.**									
Corneal decompensation	4	1	0.20	0	1	1.0	2	0	1.0
Phthisis	0	9	0.003	0	7	0.13	0	0	n/a
Hypotony	2	4	1.0	0	2	1.0	2	0	1.0
Uveitis	1	1	1.0	0	0	1.0	1	1	0.49
Progression to NLP	2	6	0.017	1	4	0.30	1	0	1.0

* All *p*-values were from Fisher exact test. ECP: endoscopic cyclophotocoagulation; TCP: transcleral cyclophotocoagulation; AC: anterior chamber; RD: retinal detachment. NVG: neovascular glaucoma; CME: cystoid macular edema; NLP: no light perception.
